# IKKβ binds NLRP3 providing a shortcut to inflammasome activation for rapid immune responses

**DOI:** 10.1038/s41392-022-01189-3

**Published:** 2022-10-19

**Authors:** Yaw Asare, Margarita Shnipova, Luka Živković, Christina Schlegl, Federica Tosato, Arailym Aronova, Markus Brandhofer, Laura Strohm, Nathalie Beaufort, Rainer Malik, Christian Weber, Jürgen Bernhagen, Martin Dichgans

**Affiliations:** 1 Institute for Stroke and Dementia Research (ISD), University Hospital, Ludwig-Maximilians-University (LMU), Munich, Germany; 2 Munich Cluster for Systems Neurology (SyNergy), Munich, Germany; 3 Institute for Cardiovascular Prevention (IPEK), LMU, Munich, Germany

**Keywords:** Inflammation, Innate immunity

**Dear Editor,**

A rapid immune response to signals released from pathogens and injuries is critical for maintaining tissue integrity and restoring homeostasis. This response is largely mediated by the concerted action of pattern recognition receptors (PRRs). Such cooperativity has been described for Toll-like receptors (TLRs) and NACHT, LRR, and pyrin domain-containing protein 3 (NLRP3), but the underlying molecular mechanisms remain incompletely understood. Inflammasomes are multi-protein complexes defined by a cytosolic innate immune sensor, usually a PRR, which recruits the adaptor molecule apoptosis-associated speck-like protein containing a caspase-recruitment domain (ASC) to activate the effector caspase-1 leading to the release of matured IL-1β and IL-18. Active caspase-1 further cleaves gasdermin D (GSDMD) allowing the N-terminal domain of GSDMD (GSDMD-N) to form pores in the plasma membrane, thus facilitating the release of matured IL-1β and IL-18. Pore-forming GSDMD-N further induces pyroptosis, an inflammatory form of cell death.^1^ NLRP3 inflammasome activation typically entails NF-κB-driven transcriptional priming, which in turn licenses the cell for inflammasome assembly and activation.^1^ A previously discussed paradigm of rapid inflammasome assembly without the requirement for NF-κB-driven transcriptional priming involves simultaneous engagement of TLRs and NLRP3.^2,3^ While increasingly recognized, the molecular mechanisms and ensuing biological effects remain largely undefined. Recent work has demonstrated a role of IKKβ in activation of the NLRP3 inflammasome by recruiting NLRP3 to the dispersed trans-Golgi network.^4^ Given the activating effect of TLR signaling on IKK and the central role of NF-κB in inflammasome signaling,^1,4^ we scrutinized whether IKK could directly activate the inflammasome on top of its effects on priming.

To systematically study the role of IKKβ in inflammasome activation beyond transcriptional priming, we assessed possible interactions between IKK and the inflammasome. Co-immunoprecipitation experiments in transfected HEK293 cells revealed binding of IKKβ to NLRP3 but not NLRC4, NLRP1, and AIM2 (Fig. 1a, b). To determine the specific domains of NLRP3 interacting with IKKβ, we co-transfected HA-IKKβ with Flag-tagged full-length or mutant NLRP3 and subjected the lysate to immunoprecipitation. We found that IKKβ binds to full-length as well as mutant NLRP3 lacking the amino-terminal pyrin domain, the singly expressed NACHT and LRR domains, but not the PYD (Fig. 1c, d). Next, we examined whether the enzymatic activity of IKKβ is required for this interaction and found that the wild-type, constitutively active, and catalytically inactive IKKβ all bind to NLRP3 (Supplementary Fig. 1a). An appreciable proportion of NLRP3 was found in IKKβ precipitates and vice versa (Supplementary Fig. 1b, c). As demonstrated by confocal microscopy, IKKβ and NLRP3 colocalized in bone marrow-derived macrophages (BMDMs) transfected with HA-IKKβ and stimulated with LPS and nigericin (Fig. 1e). Importantly, IKKβ promoted the oligomerization of NLRP3 (Fig. 1f), possibly in part through effects on the recruitment of NLRP3 to the dispersed TGN. ^4,5^ To further analyze this interaction at endogenous levels and determine the effect of inflammasome activation on this interaction, we treated human THP-1 macrophages with LPS and NLRP3 activators (nigericin or ATP) and subjected the lysate to immunoprecipitation. Consistent with the results in HEK293 cells, we found that IKKβ binds to NLRP3 at endogenous levels in THP-1 macrophages stimulated with LPS and NLRP3 activators or left unstimulated, indicating the constitutive nature of this interaction (Fig. 1g). We found no binding of p38 MAP Kinase to NLRP3 (Supplementary Fig. 1d), further supporting the specificity of the identified interactions. As a last step to corroborate this interaction, we performed microscale thermophoresis (MST) analysis by titrating MST-Red-NLRP3 against increasing concentrations of IKKβ. The obtained curve revealed a direct interaction between IKKβ and NLRP3 and a *K*_D_ value of 1.43 ± 0.35 µM was derived for the binding affinity (Fig. 1h). Collectively, these findings identify IKKβ as an interactor of NLRP3 promoting its oligomerization.

**Fig. 1:**
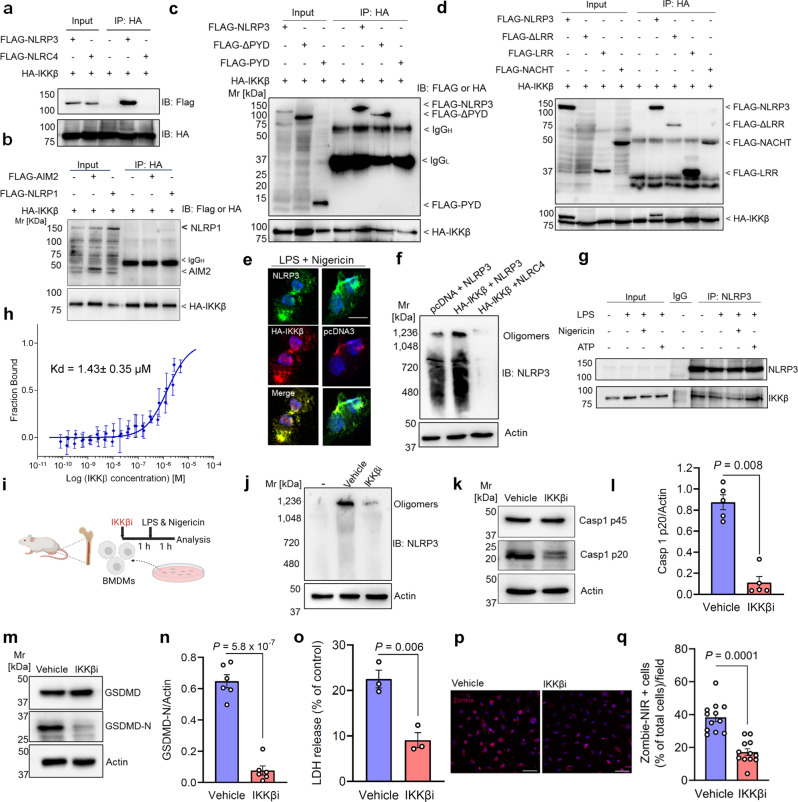
IKKβ binds NLRP3 to induce rapid inflammasome assembly and pyroptotic cell death. **a**–**d** HEK293 cells were transiently cotransfected with HA-tagged full-length IKKβ, Flag-tagged full-length or mutant NLRP3, and Flag-tagged full-length NLRC4, AIM2, or NLRP1. Shown are representative immunoblots depicting the binding of IKKβ to NLRP3, but not NLRC4, AIM2, and NLRP1 (**a**, **b**), and specific domains of NLRP3 (**c**, **d**). *n* = 3 to 4 independent experiments. **e** Determination of colocalization between IKKβ and NLRP3 in BMDMs by confocal microscopy. Shown are representative immunostainings of three independent experiments. Scale bar = 20 µm. **f** Representative immunoblot showing the assessment of NLRP3 oligomerization in transfected HEK293 cells stimulated with nigericin and analyzed by blue native PAGE. *n* = 3 independent experiments. **g** THP-1 macrophages were either stimulated with LPS (200 ng/mL) for 4 h and nigericin (5 µM) for 60 min or ATP (5 mM) for 30 min or left unstimulated. Shown is a representative immunoblot depicting the endogenous binding of IKKβ to NLRP3. *n* = 3 independent experiments. **h** Protein–protein interactions between IKKβ and NLRP3 were analyzed in solution applying microscale thermophoresis (MST). 50 nM MST-Red-NLRP3 was titrated against increasing concentrations of IKKβ. Plotted is the fraction of bound MST-Red-NLRP3 (fraction bound) over the indicated concentrations of IKKβ (log scale). Data are represented as mean ± SD, with 3–4 data points for each concentration of IKKβ. Two independent titrations measured in two separate sets of capillaries. **i**–**q** Simultaneous engagement of TLRs and NLRP3. BMDMs were treated with TPCA-1 (500 nM) for 1 h and were simultaneously stimulated with LPS (200 ng/mL) and nigericin (5 µM) for 1 h. **i** Experimental outline. **j** Representative immunoblot of NLRP3 Oligomerization. *n* = 4 independent experiments. **k** Representative immunoblot depicting caspase-1 cleavage. **l** Quantification of cleaved caspase-1 normalized to actin. *n* = 5 independent experiments. **m** Representative immunoblots of GSDMD cleavage. **n** Quantification of GSDMD cleavage normalized to actin. *n* = 6 independent experiments. **o** Measurement of LDH release. *n* = 3 independent experiments. **p** Determination of Zombie uptake. Shown are representative immunostainings of three independent experiments. Scale bar = 50 µm. **q** Quantification of Zombie uptake. Data are represented as mean ± SEM.Two-sided unpaired t test was used in the statistical analyses after testing for normality with Shapiro–Wilk-Test

To detail the consequence of this interaction, we pharmacologically inhibited IKKβ with TPCA-1 in BMDMs. The dose of TPCA-1 used was chosen after titration (Supplementary Fig. 2a–e). Quantitative proteomic profiling of IKKβ-inhibited BMDMs revealed a perturbation of proteins related to signaling in the immune system and pyroptosis including Gsdmdc1, Dhx9, Cycs, and Casp8 (Supplementary Fig. 3a–d). Given this evidence and our data indicating that IKKβ binds NLRP3, we employed two treatment and stimulation paradigms to assess the effects of IKKβ inhibition on NLRP3 inflammasome activation. The first paradigm involved inhibition of IKKβ before LPS priming and nigericin stimulation (pre-priming inhibition), while the second involved LPS priming followed by IKKβ inhibition and subsequent nigericin activation (post-priming inhibition). The latter served as an attempt to examine the direct effect of IKKβ on inflammasome activation rather than priming. Post-priming inhibition of IKKβ in BMDMs resulted in reduced cleavage of caspase-1 and GSDMD (Supplementary Fig. 4a–e), which went along with reduced maturation and secretion of IL-1β and IL-18 (Supplementary Fig. 4f, g) and limited pyroptosis in macrophages as revealed by reduced LDH release (Supplementary Fig. 4h). Similar observations were made for pre-priming inhibition, when cells were treated with either TPCA-1 or siRNA-mediated knockdown of IKKβ (Supplementary Fig. 2f and 5a–i).

Next, we investigated the involvement of IKKβ in rapid assembly of the NLRP3 inflammasome by challenging cells simultaneously with LPS and nigericin for 60 min (Fig. 1i). Inhibition of IKKβ limited rapid assembly of the inflammasome as demonstrated by reduced NLRP3 oligomerization (Fig. 1j). Immunostaining with anti-caspase-1 and anti-ASC antibodies revealed caspase-1 and ASC in a high molecular-mass NLRP3 complex. Pharmacological inhibition of IKKβ limited the formation of this large oligomeric complex (Supplementary Fig. 6a, b). IKKβ inhibition further decreased caspase-1 activation (Fig. 1k, l), reduced IL-18 release (Supplementary Fig. 5j, k), and GSDMD cleavage (Fig. 1m, n). This restricted the formation of pore-forming GSDMD-N to induce pyroptosis in macrophages as demonstrated by reduced LDH release (Fig. 1o) and decreased uptake of Zombie NIR, an independent readout of pyroptosis (Fig. 1p, q). Hence, aside from effects on transcriptional priming, IKKβ further bridges TLR and NLR signaling providing a “shortcut” to inflammasome activation for prompt immune response.

Taken together, the results presented here show that (i) IKKβ directly interacts with NLRP3; (ii) IKKβ promotes the oligomerization of NLRP3; and (iii) pharmacological inhibition of IKKβ reduces rapid caspase-1 activation and limits GSDMD cleavage to restrict macrophage pyroptosis. Our findings thus provide a mechanistic explanation for rapid immune responses induced by the TLR-NLR signaling axis and implicate IKKβ as an essential regulator of pyroptotic cell death. This should be accounted for when designing therapeutic strategies that block TLR-NLR signaling in order to avoid increased rates of infection as recently seen in therapeutic neutralization of IL-1β.^6^

## Supplementary information

Supplementary Material

## Data Availability

All data and materials are presented in the main manuscript or supplementary materials and are available on request. The mass spectrometry proteomics data have been deposited to the ProteomeXchange Consortium via the PRIDE partner repository with the dataset identifier PXD036118.
