# Supplementary material for: IKKβ binds NLRP3 providing a shortcut to inflammasome activation for rapid immune responses
Source: Signal Transduct Target Ther. 2022 Oct 19;7:355. doi: 10.1038/s41392-022-01189-3 (PMC9579124; doi:10.1038/s41392-022-01189-3)
Supplement: Supplementary file 1 — Supplementary Material [file 41392_2022_1189_MOESM1_ESM.docx]

**Supplementary Materials**

IKKβ binds NLRP3 providing a shortcut to inflammasome activation for rapid immune responses

Yaw Asare^1^*, Margarita Shnipova^1^, Luka Živković^1^, Christina Schlegl^1^, Federica Tosato^1^, Arailym Aronova^1^, Markus Brandhofer^1^, Laura Strohm^2^, Nathalie Beaufort^1^, Rainer Malik^1^, Christian Weber^2,3^, Jürgen Bernhagen^1,2^, Martin Dichgans^1, 2^*

^1^Institute for Stroke and Dementia Research (ISD), University Hospital, Ludwig-Maximilians-University (LMU), Munich, Germany; ^2^Munich Cluster for Systems Neurology (SyNergy), Munich, Germany; ^3^ Institute for Cardiovascular Prevention (IPEK), LMU

* Correspondence to: martin.dichgans@med.uni-muenchen.de & yaw.asare@med.uni-muenchen.de

This PDF file includes:

Materials and Methods

Supplementary Figures. S1 to S6

***Materials and Methods***

**Reagents**

All NLRP3 constructs were a kind gift from Dr. Florian Schmidt and NLRC4 construct was received from Dr. Veit Hornung. Human IKKβ-HA (pUNO1-hIKBKB-HA3x) was purchased from *in vivo* gene. FLAG-IKKbeta (S177E, S181E)-pcw107 was a gift from David Sabatini & Kris Wood (Addgene plasmid # 64609)^1^ while IKKβ K44M was a gift from Anjana Rao (Addgene plasmid # 11104)^2^. HA-Tag (C29F4) #3724, GSDMD (E9S1X) #39754, and IKKβ (D30C6) #8943 antibodies were purchased from Cell Signalling. NLRP3 (Cryo-2) and anti-Caspase 1 (p20) were from Adipogen. Anti-Flag M2 antibody (F1804) was from Sigma. ON-TARGETplus Mouse Ikbkb siRNA and ON-TARGETplus Non-targeting Control Pool were purchased from Horizon.

**Generation of bone marrow-derived macrophages and THP1 macrophages**

Mouse bone marrow-derived macrophages (BMDMs) were generated as established ^3^. In brief, bone marrow cells were flushed from the femur and tibiae with PBS, filtered through 40-µm cell strainer, and cultured in RPMI medium supplemented with 15% L929-conditioned medium. Stimulations with LPS and nigericin were performed in serum-free medium. For generation of THP1 macrophages, THP1 monocytes were cultured with 100 nM PMA for 72 h before stimulating the cells with LPS, nigericin, or ATP for the endogenous interactions.

**Cell lysis, co-immunoprecipitation and Western blot analysis**

For total cell lysates, cells were washed with cold PBS and lysed either directly with 1x NuPAGE-LDS-sample buffer (Invitrogen) containing 1 mmol/l DTT (Sigma Aldrich) or with 1x cell lysis buffer (#9803, Cell signaling) containing 20 mM Tris-HCl (pH 7.5), 150 mM NaCl, 1 mM Na_2_EDTA, 1 mM EGTA, 1% Triton, 2.5 mM sodium pyrophosphate, 1 mM beta-glycerophosphate, 1 mM Na_3_VO_4,_ 1 µg/mL leupeptin. Protease and phosphatase inhibitors (Roche) were added to all buffers. For immunoprecipitation experiments, HEK293 cells were transiently co-transfected with HA-tagged full-length or mutants of IKKβ and Flag-tagged full-length or mutants of NLRP3 (ΔPYD, LRR, NACHT, PYD, and ΔLRR domains). Further co-transfections involved the use of Flag-NLRC4, Flag-AIM2, HA-IKKα, and HA-IKKγ. Endogenous interactions were performed in THP1 macrophages stimulated with LPS (200 ng/ml) for 4 h and nigericin (5 µm) for 1 h or ATP (5 mM) for 30 min or left unstimulated. Cell lysates were precleared with Protein A or G beads. Following incubation of lysates with primary antibodies (anti-Flag, anti-HA, or anti-NLRP3) overnight at 4°C, the ensuing protein complexes were incubated with protein A or G beads for 2 h. Antibody-antigen complexes were eluted from the beads after washing three times with 1x cell lysis buffer containing 150 mM NaCl and a final stringent wash with 250 mM NaCl for two times. Total cell lysates or coimmunoprecipitated proteins were separated by SDS-PAGE, transferred to a PVDF membrane (Bio-Rad), and detected with the appropriate antibodies. Primary antibodies were incubated overnight at 4⁰C. HRP-conjugated anti-mouse or anti-rabbit antibodies were used as secondary antibodies and blots were developed with Immobilon Western HRP Substrate (Merck Millipore). Protein bands were visualized with a Fusion Fx7 and quantified using Image J 1.47v software (Wayne Rasband).

**Microscale thermophoresis (MST)**

Direct protein-protein interactions between IKKβ and fluorescently labeled NLRP3 were analyzed by microscale thermophoresis on a Monolith NT.115 instrument equipped with green/red filters (NanoTemper Technologies GmbH, Munich, Germany). For labeling of recombinant human NLRP3 (OriGene Technologies, Inc, Rockville, USA), the Monolith Protein Labeling Kit RED-NHS 2^nd^ Generation from NanoTemper (Munich, Germany) was used, following the manufacturer’s instructions. Experiments were performed essentially following a previously described protocol ^4,5^. Measurements were performed in standard NanoTemper Monolith capillaries at 25°C with 80% MST power and 95% LED excitation power to obtain an initial fluorescence count in the range of 180 to 240. MST traces were recorded for 40 s (-5 s to +35 s), at default settings, with the sample being heated between 0 s and 30 s. All measurements were performed in MST assay buffer (50 mM Tris-HCl, pH 7.4, 150 mM NaCl, 10 mM MgCl_2_, 0.05% Tween-20). MST-Red-NLRP3 was used at a fixed concentration of 50 nM and mixed with equal volumes of serial dilutions of recombinant human IKKβ (ActiveMotif, Waterloo, Belgium), resulting in a final NLPR3 concentration of 25 nM. Prior to loading capillaries for measurements, the prepared samples were incubated for at least 40 min on ice. Three to four MST traces per IKKβ concentration, prepared in two individual serial dilutions each, were analyzed according to the K_D_ model using the default settings, analyzing the temperature related intensity change (TRIC) of the fluorescent label (“cold region” from -1 to 0 s, “hot region” from 0.5 to 1.5 s) and using the MO.AffinityAnalysis V2.3 software (NanoTemper Technologies). Curve fitting for data representation was performed by GraphPad Prism Version 6.07 (‘one site – total binding’) based on data exported from MO.AffinityAnalysis software.

**ELISA**

For maturation and release of Il-1β and Il-18, BMDMs were stimulated with LPS and nigericin. Levels of Il-1β and Il-18 were measured in supernatants using commercially available ELISA kits (R&D systems).

**LDH release assay**

LDH release was determined in supernatants of BMDMs stimulated with LPS and nigericin. The assay was performed using the Pierce LDH cytotoxicity assay kit following manufacturer’s instructions (Thermo Fisher Scientific). LDH release was calculated as LDH release [%] = (LDH activity in stimulated cells – spontaneous LDH activity in unstimulated cells) / (Maximum LDH activity after complete lysis – spontaneous LDH activity) × 100.

**Zombie uptake assay**

Uptake of Zombie dye, a second independent methodology to assay membrane integrity, was assessed in BMDMs stimulated with LPS and nigericin following manufacturer’s instructions (Biolegend). Cells were washed with 1× PBS and fixed with 4% paraformaldehyde-PBS solution for 10 minutes. Nuclei was counterstained with 4,6-diamidino-2-phenylindol and images acquired with 40× oil objective.

**Confocal Microscopy**

BMDMs were transfected with HA-IKKβ using electroporation. Cells were allowed to recuperate and either stimulated with LPS and nigericin or left unstimulated. Following washing with 1× PBS, cells were fixed with 4% paraformaldehyde-PBS solution for 10 minutes, and permeabilized using 0.1% Triton X. Cells were then blocked for 1 hour with 0.2% FCS, 0.2% BSA, and 0.002% fish skin gelatin in 1× PBS. Primary antibodies against NLRP3 and HA were incubated overnight at 4°C. 4,6-diamidino-2-phenylindol as well as Cy3 and Alexa Fluor 488–labeled secondary antibodies were incubated for 1 hour at room temperature. Cells were washed and sealed with a coverslip coated in fluoromount mounting medium (Sigma). Imaging was performed with the confocal microscope (LSM 880, Zeiss) using the 40× oil objective and analyzed with the ZEN software (Zeiss).

**Blue native PAGE**

Blue native gel electrophoresis was performed using the Bis-Tris Native PAGE system as previously described ^6,7^. 2.5×10^6^ HEK293 cells were plated in 60 mm dishes and transfected with 2 µg each of the indicated plasmids. Following transfections, the cells were stimulated with 5 μM nigericin and washed once with cold PBS and lysed in ice-cold native lysis buffer (20 mM Bis-tris, 500mM ε-aminocaproic acid, 20 mM NaCl, 10% (w/v) glycerol, 0.5% digitonin, 0.5 mM Na_3_VO_4_, 1mM PMSF, 0.5mM NaF, 1× EDTA-free Roche protease inhibitor cocktail, pH 7.0) for 15 min on ice. Cell lysates were clarified by centrifugation at 18.000g for 30 min at 4°C and analysed without further purification steps. Total proteins were quantified using the Pierce BCA protein assay (#23225, ThermoFisher).

For experiments with primary macrophages, 4.5×10^6^ BMDMs were seeded in 10 cm dishes. On the following day, cells were serum-starved for 1 h and then treated with 500 nM TPCA-1 or DMSO for 1 h. Subsequently, the cells were stimulated with 5 μM nigericin and 200 ng/ml LPS for 1 h. Cell lysis was performed as above. Samples were separated on 3-12% blue native PAGE (#BN1001BOX, Invitrogen). Native gels were incubated in 10% SDS solution for 5 min before transfer to PVDF membranes (Millipore), followed by conventional western blotting.

**Proteomics analysis**

BMDMs treated with TPCA-1, were stimulated with TNF-α (50 ng/mL) for 24 h. TNF-α, implicated in priming of the inflammasome^8^, was used as an endogenous pro-inflammatory stimulus and an inducer of sterile inflammation. Thereafter cells were collected and lysed in urea buffer (9 M Urea, 50 mM Tris pH 8, 150 mM NaCl, 1x Roche protease inhibitor cocktail) followed by short sonification. Samples were cleared by centrifugation and protein amounts were adapted. Protein reduction was performed with dithiothreitol (DTT; 5 mM final) for 25 min at 56°C and protein alkylation by the addition of iodoacetamide (14 mM final) for 30 min at room temperature. Protein mixtures were quenched with DTT and diluted 1:5 with 1 M Tris-Hcl, pH 8.2. For increased peptide recovery, proteins were digested at room temperature for 3 h with LysC (FUJIFILM, 2 µl/100 µg protein) before overnight tryptic digest at 37°C (0.5 µg/100µg protein). The following day, digestion was stopped with 10% TFA. To increase analysis depth, samples were pre-fractioned by a C18-SCX custom-made stage tip^9^. Fractions were eluted stepwise with increasing NH4AcO concentrations (20 mM to 500 mM) and desalted on a separate C18 stage tip. Desalted peptides were loaded on a custom-made 75 mm x 15 cm fused silicia capillary filled with C18-AQ resin (Reprosil Pur 120, 1.9 µm, Dr. Maisch HPLC) using an Easy-nLC1200 liquid chromatography. Samples were separated for 75 min with a 5-95% ACN gradient in 0.5% acetic acid using a Q Exactive HF mass spectrometer (Thermo Scientific). MS raw data were processed with MaxQuant (version 1.6.0.1). The mass spectrometry proteomics data have been deposited to the ProteomeXchange Consortium via the PRIDE^10^ partner repository with the dataset identifier PXD036118. Intensities were log2-transformed and normalized using variance stabilizing normalization (vsn). Relative quantification and statistical analysis were performed for all proteins identified by at least 2 peptides in at least two samples per group. Pyroptosis-related proteins were identified using Uniprot keywords (https://www.uniprot.org). Pathway enrichment analyses were performed using DAVID (https://david.ncifcrf.gov).

**Statistical analysis**

Statistical analyses were performed with Graphpad Prism 9 using two-tailed unpaired T-test or Mann-Whitney test as appropriate after normality testing with Shapiro-Wilk-Test. One-way Anova with Holm-Sidak’s or Sidak’s multiple comparisons were used as appropriate. Sample sizes were chosen based on experience from previous studies^3,11^.

**Reference**

1 Martz, C. A. *et al.* Systematic identification of signaling pathways with potential to confer anticancer drug resistance. *Sci Signal* **7**, ra121, (2014).

2 Mercurio, F. *et al.* IKK-1 and IKK-2: cytokine-activated IkappaB kinases essential for NF-kappaB activation. *Science* **278**, 860-866, (1997).

3 Asare, Y. *et al.* Histone Deacetylase 9 Activates IKK to Regulate Atherosclerotic Plaque Vulnerability. *Circ Res* **127**, 811-823, (2020).

4 Brandhofer, M. *et al.* Heterocomplexes between the Atypical Chemokine MIF and the CXC-Motif Chemokine CXCL4L1 Regulate Inflammation and Thrombus Formation. *bioRxiv*, (2021).

5 Kontos, C. *et al.* Designed CXCR4 mimic acts as a soluble chemokine receptor that blocks atherogenic inflammation by agonist-specific targeting. *Nat Commun* **11**, 5981, (2020).

6 He, Y., Zeng, M. Y., Yang, D., Motro, B. & Nunez, G. NEK7 is an essential mediator of NLRP3 activation downstream of potassium efflux. *Nature* **530**, 354-357, (2016).

7 Swamy, M., Siegers, G. M., Minguet, S., Wollscheid, B. & Schamel, W. W. Blue native polyacrylamide gel electrophoresis (BN-PAGE) for the identification and analysis of multiprotein complexes. *Sci STKE* **2006**, pl4, (2006).

8 McGeough, M. D. *et al.* TNF regulates transcription of NLRP3 inflammasome components and inflammatory molecules in cryopyrinopathies. *J Clin Invest* **127**, 4488-4497, (2017).

9 Rappsilber, J., Mann, M. & Ishihama, Y. Protocol for micro-purification, enrichment, pre-fractionation and storage of peptides for proteomics using StageTips. *Nat Protoc* **2**, 1896-1906, (2007).

10 Perez-Riverol, Y. *et al.* The PRIDE database resources in 2022: a hub for mass spectrometry-based proteomics evidences. *Nucleic Acids Res* **50**, D543-D552, (2022).

11 Asare, Y. *et al.* Inhibition of atherogenesis by the COP9 signalosome subunit 5 in vivo. *Proc Natl Acad Sci U S A* **114**, E2766-E2775, (2017).

**Supplementary Figures**


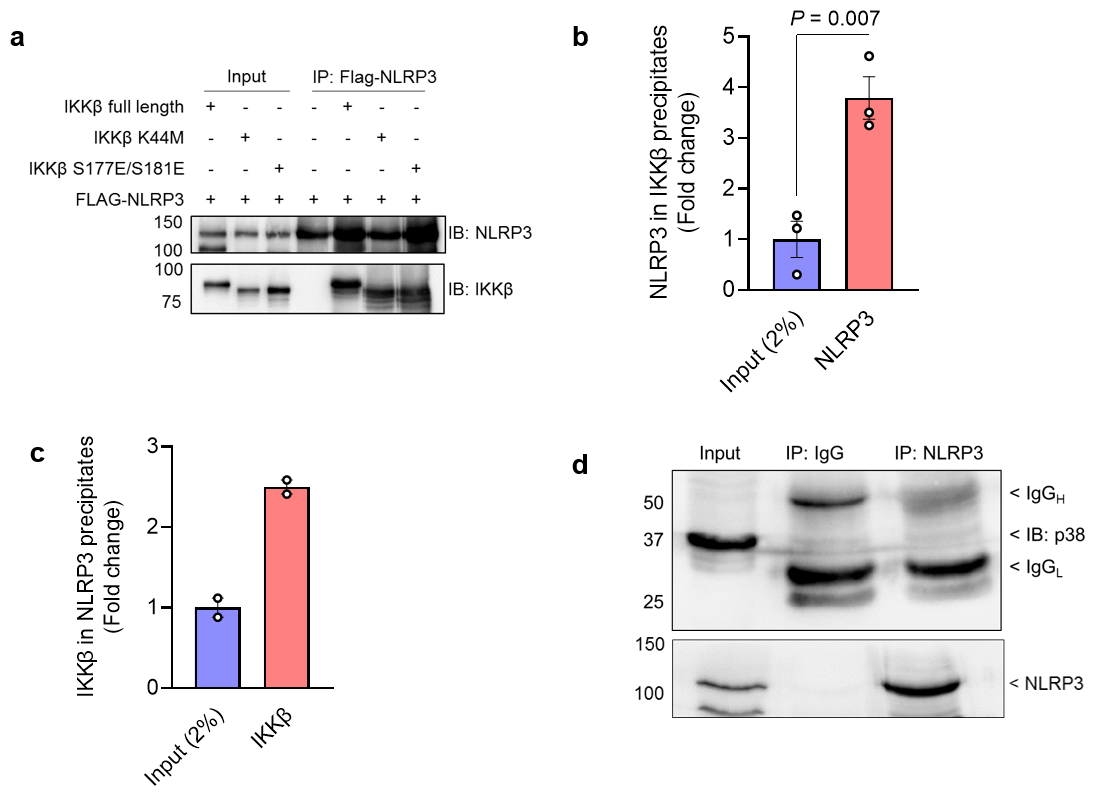


**Figure.** **S1**. **Binding of IKKβ to NLRP3 and effects on oligomeric complex formation**. (**a-c**) HEK293 cells were transiently cotransfected with full-length or mutant IKKβ and Flag-tagged full-length NLRP3. **a**) Shown are representative immunoblots depicting interaction between full-length or mutant IKKβ and NLRP3 upon immunoprecipitation of Flag-NLRP3. n=2 independent experiments. (**b**) Quantification of the proportion of NLRP3 in IKKβ precipitates. n=3 independent experiments. (**c**) Quantification of the proportion of IKKβ in NLRP3 precipitates. n=2 independent experiments. (**d**) Representative immunoblot of p38 and NLRP3 upon immunoprecipitation of NLRP3 in THP-1 macrophages. n=3 independent experiments.


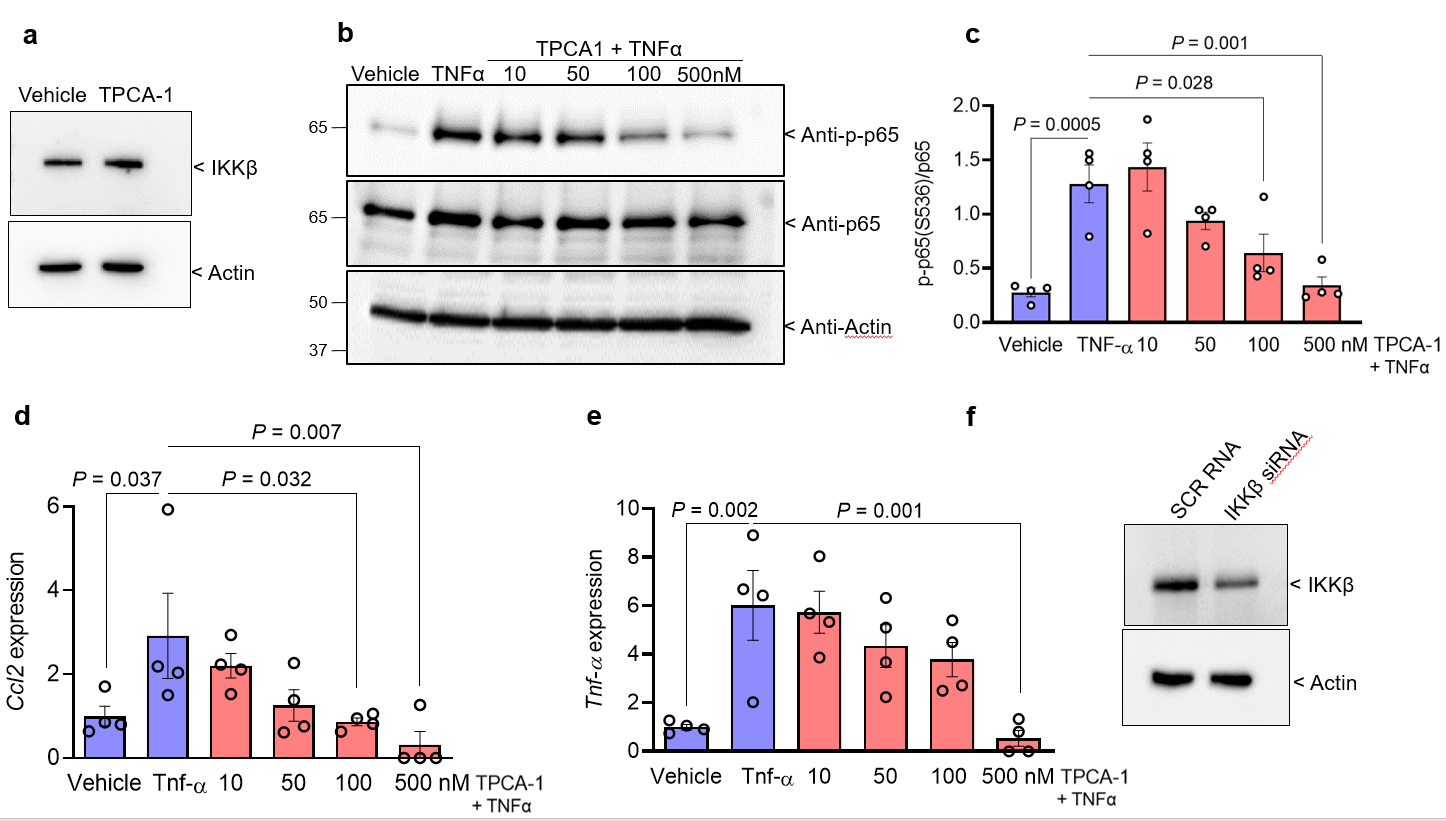


**Figure.** **S2. Pharmacological inhibition and siRNA-mediated knockdown of IKKβ in Mφ**. (**a-e**) BMDMs were pre-treated with different concentrations of TPCA-1 or vehicle for 1 h and were stimulated with TNF-α. (**a**) Representative immunoblots of IKKβ and actin in non-stimulated cells pre-treated with 500 nM TPCA-1. n=3 independent experiments. (**b**) Representative immunoblots of P-p65, p65, and actin. (**c**) Quantification of immunoblots in B. n=4 independent experiments. (**d, e**) Gene expression of *Ccl2* and *Tnf-α*. n=4 independent experiments. (**f**) Representative immunoblot of siRNA-mediated IKKβ depletion in BMDMs. n=3 independent experiments. One-way Anova with Holm-Sidak’s or Sidak’s multiple comparisons were used in the statistical analyses.


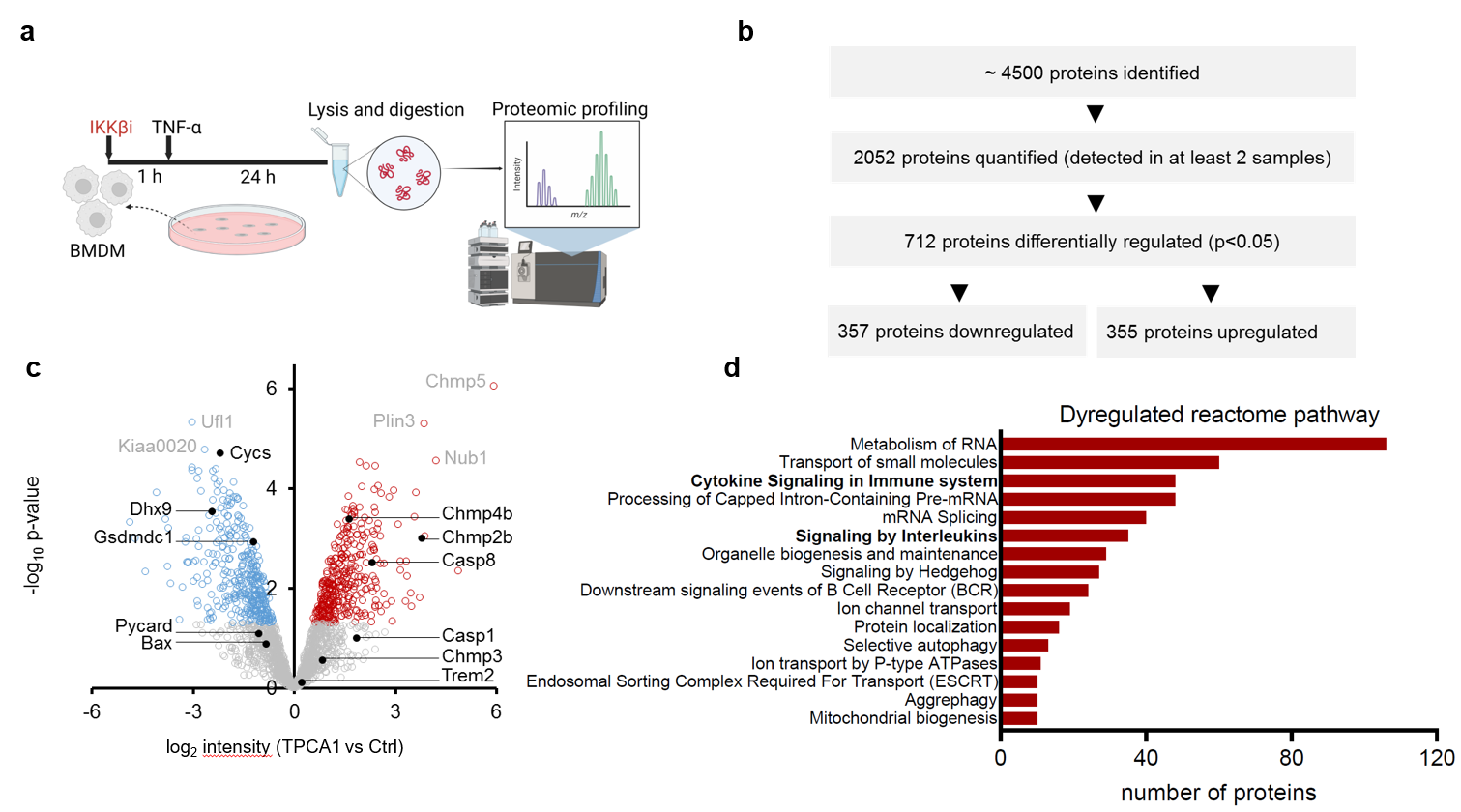


**Figure.** **S3. Proteomic profiling of IKKβ-inhibited BMDMs reveals a signature for perturbation of proteins involved in immune system signaling and pyroptosis.** BMDMs were treated with TPCA-1 (500 nM; designated IKKβi) or vehicle for 1 h and stimulated with TNF-α (50 ng/mL) for 24 h. Following lysis and digestion, the cellular proteome was resolved by LC-MS/MS and label-free quantification (LFQ). (**a**) Experimental outline. (**b**) Flowchart of proteins identified, quantified and differentially regulated. (**c**) Volcano plot of log_2_ intensity ratio (TPCA1 vs. control) and −log_10_ p values of all quantified proteins (n = 4 mice per condition). Red and blue circles represent proteins with a higher and a lower abundance respectively (p-value < 0.05). Proteins involved in pyroptosis are highlighted in black. Proteins are labelled by gene name. (**d**) Reactome pathway showing the signature of differentially regulated proteins (p value < 0.05**).**


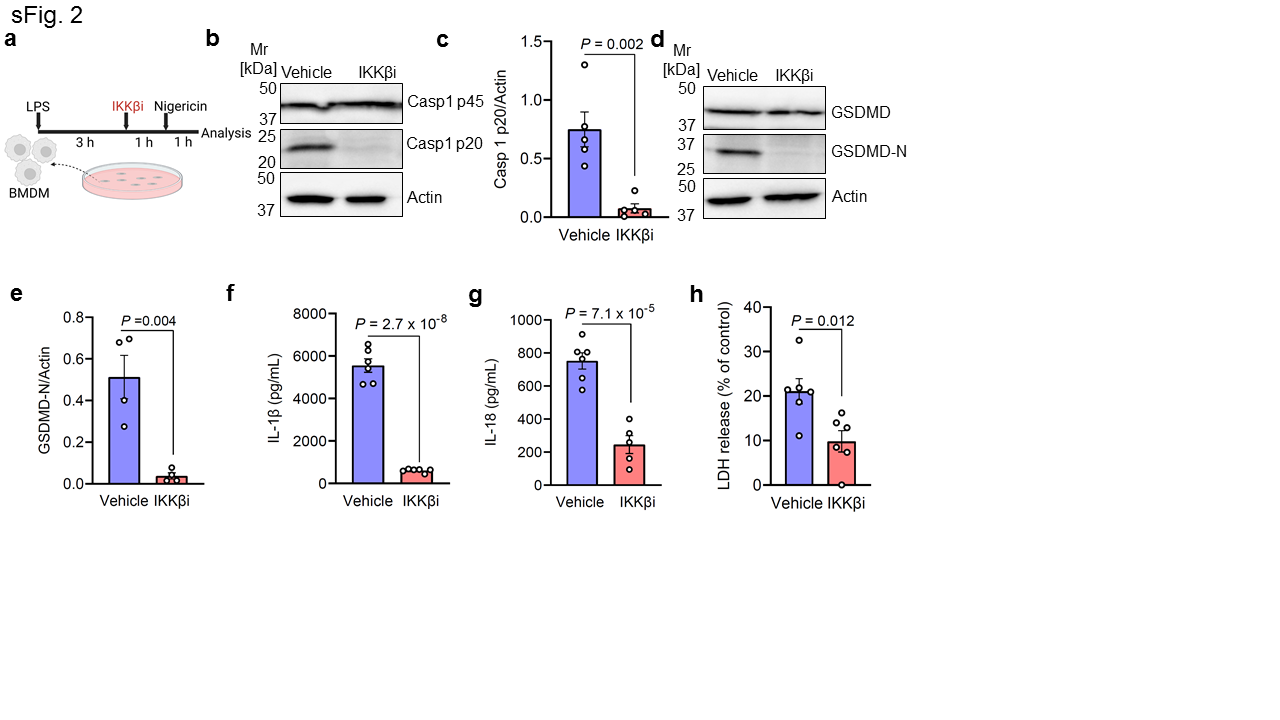
**Figure.** **S4. Post-priming inhibition of IKKβ reduces inflammasome activation to limit pyroptosis in macrophages**. (**a-h**) Post-priming inhibition. BMDMs were stimulated with LPS (100 ng/mL) for 3 h and treated with TPCA-1 (500 nM, designated IKKβi) for 1 h prior to nigericin (5 µM) stimulation for 1 h. (**a**) Experimental outline. (**b**) Representative immunoblots of caspase-1 cleavage. (**c**) Quantification of cleaved caspase-1 normalized to actin. n=5 independent experiments. (**d**) Representative immunoblots of gasdermin D cleavage. (**e**) Quantification of gasdermin D normalized to actin. n=4 independent experiments. (**f**) Quantification of IL-1β and (**g**) IL-18 release. n = 5-6 independent experiments. (**h**) Measurement of LDH release. n = 6 independent experiments.


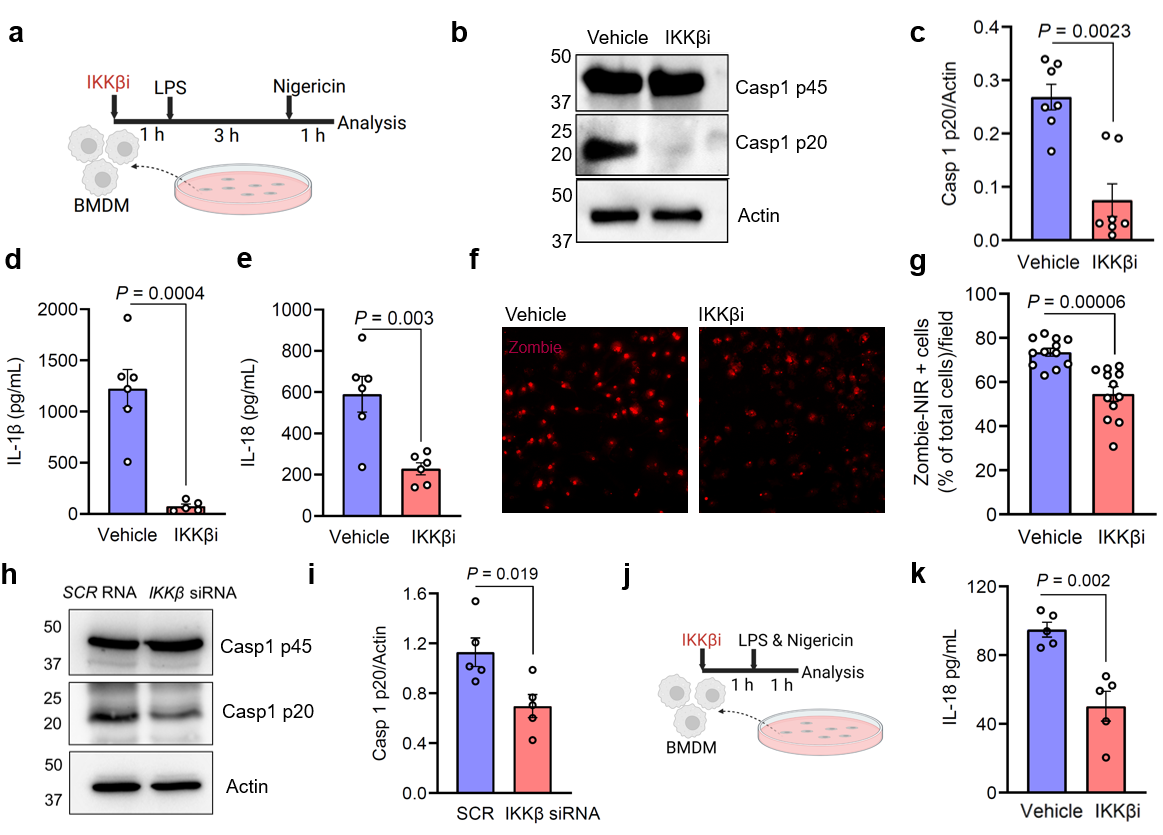


**Figure.** **S5. Effects of Pre-priming inhibition of IKKβ on inflammasome activation**. (**a-g**) Pre-priming inhibition. BMDMs were treated with TPCA-1 (500 nM; designated IKKβi) for 1 h and then stimulated with LPS (100 ng/mL) for 3 h and nigericin (5 µM) for 1 h. (**a**) Experimental outline. (**b**) Representative immunoblots of caspase-1 cleavage. (**c**) Quantification of cleaved caspase-1 normalized to actin. n=7 independent experiments. (**d**) Quantification of IL-1β and (**e**) IL-18 release. n = 5-6 independent experiments. (**f**) Determination of Zombie uptake. Shown are representative immunostainings of 3 independent experiments. (**g**) Quantification of Zombie uptake as a readout for pyroptosis. (**h**, **i**) siRNA-mediated knockdown of IKKβ in BMDMs. (**h**) Representative immunoblot of caspase-1 cleavage. (**i**) Quantification of cleaved caspase-1. n=5 independent experiments. (**j, k**) Simultaneous engagement of TLRs and NLRP3. BMDMs were treated with TPCA-1 (500 nM) for 1 h and were simultaneously stimulated with LPS (200 ng/mL) and nigericin (5 µM) for 1 h. (**j**) Experimental outline. (**k**) Quantification of IL-18 release. n=5 independent experiments. Two-sided unpaired t test was used in all after testing for normality with Shapiro-Wilk-Test except in C where Mann-Whitney test was used.


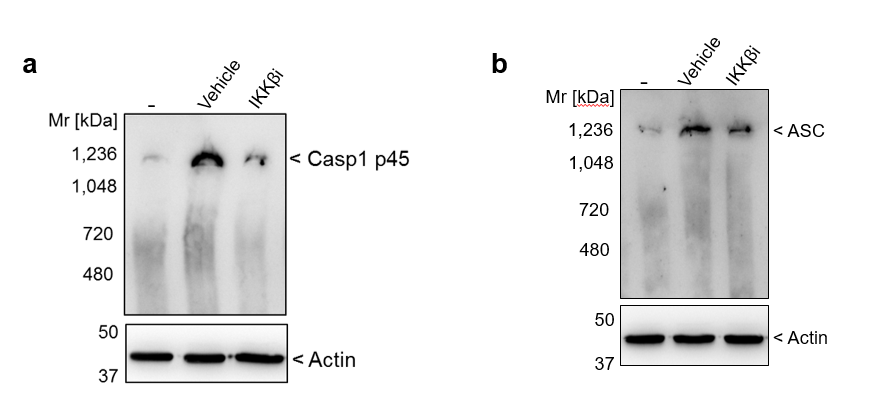


**Figure.** **S6. Presence of caspase-1 and ASC in high molecular-mass NLRP3 complex** (**a**, **b**) Simultaneous engagement of TLRs and NLRP3. BMDMs were treated with TPCA-1 (500 nM; designated IKKβi) for 1 h and were simultaneously stimulated with LPS (200 ng/mL) and nigericin (5 µM) for 1 h. Representative immunoblots of caspase-1 (**a**) and ASC detection (**b**) in high molecular-mass NLRP3 complex. n=3 independent experiments.
